# Lactoferrin Retargets Human Adenoviruses to TLR4 to Induce an Abortive NLRP3-Associated Pyroptotic Response in Human Phagocytes

**DOI:** 10.3389/fimmu.2021.685218

**Published:** 2021-05-20

**Authors:** Coraline Chéneau, Karsten Eichholz, Tuan Hiep Tran, Thi Thu Phuong Tran, Océane Paris, Corinne Henriquet, Jeffrey J. Bajramovic, Martine Pugniere, Eric J. Kremer

**Affiliations:** ^1^ Institut de Génétique Moléculaire de Montpellier, Université de Montpellier, CNRS, Montpellier, France; ^2^ Institut de Recherche en Cancérologie de Montpellier, INSERM, Université Montpellier, Institut Régional du Cancer, Montpellier, France; ^3^ Alternatives Unit, Biomedical Primate Research Centre, Rijswijk, Netherlands

**Keywords:** Lactoferrin, adenovirus, dendritic cell (DC), TLR4 (Toll-like receptor 4), IL-1β

## Abstract

Despite decades of clinical and preclinical investigations, we still poorly grasp our innate immune response to human adenoviruses (HAdVs) and their vectors. In this study, we explored the impact of lactoferrin on three HAdV types that are being used as vectors for vaccines. Lactoferrin is a secreted globular glycoprotein that influences direct and indirect innate immune response against a range of pathogens following a breach in tissue homeostasis. The mechanism by which lactoferrin complexes increases HAdV uptake and induce maturation of human phagocytes is unknown. We show that lactoferrin redirects HAdV types from species B, C, and D to Toll-like receptor 4 (TLR4) cell surface complexes. TLR4-mediated internalization of the HAdV-lactoferrin complex induced an NLRP3-associated response that consisted of cytokine release and transient disruption of plasma membrane integrity, without causing cell death. These data impact our understanding of HAdV immunogenicity and may provide ways to increase the efficacy of HAdV-based vectors/vaccines.

## Introduction

The gaps in our understanding of our innate immune response to human adenovirus (HAdV)-based vaccines are significant. For example, following vaccine injection, how do the rapid recruitment of myeloid cells, release of danger-associated molecular patterns (DAMPs), and presence of host defense proteins/peptides (HDPs), influence the response to vector-encoded transgenes? How do these variables impact HAdV uptake and efficacy?

Here we addressed the impact of an HDP on three HAdV-based vaccine vectors. HDPs, also known as antimicrobial peptides (AMPs), are evolutionarily conserved effector molecules of the innate immune system. HDPs can act directly *via* antibiotic-like properties against a broad array of infectious agents (1, 2), or indirectly by promoting the activation and maturation of antigen-presenting cells. Many HDPs are produced by neutrophils and epithelial cells of the skin, oral mucosa, and gastrointestinal tract. HDPs make up ~20% of the cytoplasmic content of neutrophils, which are among the first leukocytes to infiltrate pathogen-infected and vaccine-injected tissues (3). The rapid release of HDPs acts as part of the first line responders to the disruption of tissue homeostasis (4). Functionally, HDPs can neutralize endotoxin and recruit and modulate the activities of immune cells. The alarmins (e.g. lactoferrin, α-defensin, and cathelicidin LL-37) are a subset of HDPs that also modulate innate and adaptive immune responses by directly engaging several pathways including pattern recognition receptor (PRR) signalling in antigen-presenting cells (APCs) (1, 2). Lactoferrin, an 80 kDa multifunctional member of the transferrin family that sequesters iron, is produced primarily by neutrophils, and its physiological concentration can reach >1 mg/ml in some environments. Functionally, lactoferrin can induce dendritic cell (DC) maturation and, in the context of infections, drive Th1 responses (5–7).

In addition to their ability to directly influence innate and adaptive immune responses to bacteria, fungi, and enveloped viruses, some alarmins influence HAdV uptake (8–10). HAdVs are 150 mDa, ~90 nm diameter, nonenveloped proteinaceous particles containing a linear double-stranded DNA genome of ~36,000 (± 9,000) bp. HAdVs are classified into 7 species (A-G) and numerous types (> 100) based on serology and phylogeny. In most cases, HAdVs cause self-limiting respiratory, ocular, or gastro-intestinal tract infections in all populations regardless of health standards. Over the last 40 years the vectorization and immunogenicity of HAdVs have been of increasing interest in the context of vaccines, gene transfer, and morbidity associated with HAdV reactivation in immune-compromised individuals. The three HAdV types used in this study were chosen based on their state of development as vaccines (11–18). HAdV-C5, -D26 and -B35 are from different HAdV species, and are associated with different efficacy when used as vaccines. The *raison d’être* for the use of HAdV-D26 and -B35 is that their “low” seroprevalence (at least in European and North American cohorts) may circumvent some concerns associated with pre-existing HAdV humoral immunity (19). It is worth noting that HAdV-B35 seroprevalence is typically rare – whether this is due to the lack of infection or lack of production of type-specific antibodies is currently unknown. Attention to adenovirus immunogenicity has been further increased due to their use as vaccines during the COVID-19 pandemic (20).

In epithelial cells, alarmins influence HAdV uptake *via* multiple mechanisms. Lactoferrin acts as a bridging factor during species C HAdV (types 1, 2, 5 and 6) uptake in epithelial-like cells, independent of coxsackievirus adenovirus receptor (CAR), the primary cell surface attachment molecule for species C HAdVs (21). Adams et al. reported that lactoferrin enhances HAdV type 5 (HAdV-C5) uptake by human DCs (9) and increased maturation. However, a mechanistic understanding of how increased uptake occurs, how DC maturation is induced, and which PRRs are engaged is lacking.

In this study, we characterize the mechanism by which lactoferrin increases HAdV uptake and induces an innate response in human phagocytes. We show that lactoferrin binds HAdV-C5, -D26, and -B35 with affinities in the micromolar range and re-targets them to Toll-like receptor 4 (TLR4) complexes. TLR4-engagement induces NLRP3 inflammasome formation, release of the pro-inflammatory cytokine interleukin 1 beta (IL-1β) - but not cell death. This novel innate response is a variation of the alternative NLRP3 pathway (22). In addition to a better understanding of the immunogenicity of HAdV-based vaccines, our data resolve a discordance between the TLR4-associated response to HAdV in mice vs. that of human phagocytes (23, 24).

## Materials and Methods

### Cells and Culture Conditions

Blood samples were obtained from >100 anonymous donors at the regional blood bank (EFS, Montpellier, France). An internal review board approved the use of human blood samples. DCs were generated from freshly isolated or frozen CD14^+^ monocytes using CD14 MicroBeads human (MiltenyiBiotec) in the presence of 50 ng/ml granulocyte-macrophage colony-stimulating factor (GM-CSF) and of 20 ng/ml interleukin-4 (IL-4) (PeproTech). DCs stimulation was performed 6 days post-isolation of monocytes. Monocyte-derived Langerhans cells (LCs) were generated using 200 ng/ml GM-CSF and 10 ng/ml TGF-β. 911 and 293 E4-pIX cells were grown in Dulbecco’s modified Eagle medium (DMEM) and minimum essential medium (MEMα) with Earle’s salts, L-glutamine supplemented with 10% fetal bovine serum (FBS).

### Adenoviruses

The HAdV vectors used in this study are replication-defective (deleted in the E1 region). The HAdV-C5 vector contained a GFP expression cassette (25). The HAdV-D26 vector contained a GFP-luciferase fusion expression cassette (13). The HAdV-B35 vector contained a YFP expression cassette (26). The vectors were propagated in 911 or 293 E4-pIX cells and purified by two CsCl density gradients (25).

### DC Stimulation With HAdV-Lactoferrin Complexes

DCs (4 x 10^5^ in 400 µl of complete medium) were incubated with HAdV-C5, HAdV-D26 or HAdV-B35 (0.1 to 2 x 10^4^ physical particles (pp)/cell). We generated HAdV-lactoferrin complexes by incubating the virus with 40 µg lactoferrin (Sigma-Aldrich) for 30 min at room temperature. This corresponds to 100 µg/ml (1.25 µM) lactoferrin in 400 µl. These concentrations were chosen to reproduce those found in an inflammatory environment of infected tissues. When specified, cells were complexed with IVIG (human IgG pooled from between 5,000 and 50,000 donors/batch) (Baxter SAS) or with lactoferricin (fragment of 49 aa). Cells were incubated with HAdV-lactoferrin for 4 h, then washed and incubated again for 24 h. The TLR4 agonist lipopolysaccharide (LPS) (Sigma-Aldrich) and NLRP3 inflammasome inducer nigericin (InvivoGen) were used at 100 ng/ml and 10 µM, respectively, to induce NLRP3 inflammasome formation. The inhibitors were used at the following concentrations, TLR4 inhibitors TAK-242 (Merck Millipore) at 1 µg/ml, oxPAPC (InvivoGen) at 30 µg/ml, TRIF inhibitory peptide (InvivoGen) at 25 µM, Syk inhibitor R406 (InvivoGen) at 5 µM, KCl (Sigma-Aldrich) at 40 mM, ROS inhibitor N-acetyl-L-cysteine (Sigma-Aldrich) at 2 mM, cathepsin B inhibitor MDL 28170 (Tocris Bioscience) at 0.1 mM, NLRP3 inhibitor MCC-950/CP-456773 (Sigma-Aldrich) at 10 µM, Bay11-7082 (Sigma-Aldrich) at 10 µM, caspase-1 inhibitor WEHD (Santa Cruz) and YVAD (InvivoGen) at 20 µM, VX765 (InvivoGen) at 10 µM, caspase-8 inhibitor Z-IEDT at 20 µM, RIPK1 inhibitor GSK963 (Sigma-Aldrich) at 3 µM, RIPK3 inhibitors GSK872 (Merck Millipore) at 3 µM and necrosulfonamide (R&D systems) at 1 µM. TLR4/MD-2, TLR4 (R&D Systems), MD-2 (PeproTech) recombinant protein and anti-CD14 antibody (Beckman) were used at 20 µg/ml. Inhibitors were added on cells and recombinant proteins or antibody were added on HAdV-lactoferrin complex 1 h before stimulation.

### Surface Plasmon Resonance Analyses

SPR analyses were carried out on a BIAcore 3000 apparatus in HBS-EP buffer (10 mM HEPES, 150 mM NaCl, 3 mM EDTA, and 0.005% (v/v) polysorbate 20, pH 7.4). HAdV-C5, HAdV-D26 and HAdV-B35 diluted in acetate buffer at pH 4 were immobilized on three different flow cells of a CM5 sensor chip by amine coupling according to the manufacturer instructions. Immobilization levels were between 3,500 and 4,000 RU. Flow cell 1, without immobilized HAdV, was used as a control. Lactoferrin was injected at 100 nM on the four flow cells simultaneously. For KD determination different concentrations of lactoferrin (6.25 - 200 nM) were injected at 30 µl/min during 180 s of association and 600 s of dissociation with running buffer. Regeneration was performed with pulses of gly-HCl pH 1.7. The kinetic constants were evaluated from the sensorgrams after double-blank subtraction with BIAevaluation software 3.2 (GE Healthcare) using a bivalent fitting model for lactoferrin. All experiments were repeated at least twice for each virus on a freshly coated flow cell.

### Flow Cytometry

Cellular GFP or YFP expression from the HAdV-C5, -B35, -D26 vectors was assayed by flow cytometry. Fluorescence intensity was assessed at 24 h. TLR4 surface expression level was assessed with an anti-TLR4 antibody (Miltenyi Biotech) after 4 or 24 h. DC maturation at 24 h post-incubation was assessed by measuring CD86 surface expression level with an anti-CD86 antibody (clone 2331, BD Biosciences) or by measuring dextran (4.4 kDa) uptake (dextran TRITC, Sigma). Dextran was used at 1 mg/ml for 30 min at 37°C (or 4°C for negative control). Cells were washed and fixed with 4% PFA and fluorescence was directly analysed by flow cytometry. Cell membrane integrity was assessed by collecting cells by centrifugation 800 x g, the cell pellets were re-suspended in PBS, 2% FBS, 1 mM EDTA, 7-aminoactinomycin D (7-AAD) (Becton-Dickinson Pharmigen) and analysed on a FACS Canto II (Becton-Dickinson Pharmigen) or NovoCyte (ACEA Biosciences) flow cytometer.

Inflammasome formation was monitored as previously described (27) with minor modifications. DCs (1.5 x 10^5^ in 150 µl of complete medium) were seeded in a conical bottom 96 well plate and incubated with HDP-HAdV complexes containing 20,000 HAdV pp/cell. LPS/nigericin and immune complexed HAdV-C5 (IC-HAdV) were used as positive controls to identify inflammasome positive cells. IC-HAdV-C5 were prepared with IVIG (human IgG pooled from between 1,000 and 50,000 donors/batch) (Baxter SAS) as previously described (28). Cells were fixed by adding 50 µl 4% PFA, PBS for 10 min on ice and centrifuged at 650 x g for 5 min. Supernatants were discarded and cells were permeabilized with 150 µl PBS/3% FCS/0.1% saponin for 20 min and collected by centrifugation. Supernatant was removed, and cells were re-suspended in 100 µl 1:500 rabbit anti-ASC (N-15)-R (Santa Cruz, sc-22514-R) PBS/3%FCS/0.1% saponin and incubated overnight at 4°C. Following overnight incubation, cells were pelleted at 650 x g for 5 min, washed once with 150 µl PBS/3% FCS:0.1% saponin, pelleted again and incubated for 45 min in 100 µl 1:500 Alexa-488 1:500 donkey anti-rabbit PBS:3% FCS:0.1% saponin for 45 min at room temperature. Cells were collected again by centrifugation and re-suspended in 150 µl PBS/3% FCS/0.1% saponin. The BD FACS-Canto II was used for acquisition. Samples were gated on DC and any doublets were excluded using forward light scattering (FSC)-area versus FSC width. Inflammasome positive cells were identified in the green channel as FL1-width low, and FL1-height high.

### Cytokine Secretion

Supernatants were collected 4 or 24 h post-challenge and the levels of TNF and mature IL-1β were quantified by ELISA using OptEIA human TNF ELISA Set (BD Biosciences) and human IL-1β/IL-1F2 DuoSet ELISA (R&D systems) following the manufacturer’s instructions. In addition, 22 cytokines were detected by Luminex on Bio-plex Magpix using Bio-plex human chemokine, cytokine kit (Bio-Rad) following the manufacturer’s instructions.

### LDH Release

LDH release was quantified using an LDH Cytotoxicity Assay Kit (Thermo scientific) following the manufacturer’s instructions. Briefly, 5 x 10^5^ cells were cultures in 96-well plates, infected for 4 h, and 100 µl of supernatant were collected to assess LDH activity. Fresh reaction mixture (100 µl) was then added to each well, incubated at room temperature for 30 min, the reaction was stopped, and the absorbance was determined at 490 nm using a microplate reader (NanoQuant, Tecan).

### Quantification of mRNAs

The levels of human *TNF*, *NLRP3*, *CASP1* and *IL1B* mRNAs were analysed using quantitative reverse transcription-PCR (qRT-PCR). Total RNAs were isolated from DCs using a High Pure RNA isolation kit (Roche). Reverse transcription was performed with a Superscript III first-strand synthesis system (Invitrogen, Life Technologies) using 300 ng of total RNA and random hexamers. The cDNA samples were diluted 1:10 in water and analysed in triplicate using a LightCycler 480 detection system (Roche, Meylan, France). PCR conditions were 95°C for 5 min and 45 cycles of 95°C for 15 s, 65°C or 70°C for 15 s, and 72°C for 15 s, targeting the *GAPDH* (glyceraldehyde-3-phosphate dehydrogenase) mRNA as an internal standard. Primer sequences were as follows for ***NLRP3* [5’-CCTCTC TGATGAGGCCCAAG-3’ (NLRP3 forward) and 5’-GCAGCAAACTGGAAAGGAAG-3’ (NLRP3 reverse)] at 65°C, *IL1B* (5’-AAACAGATGAAGTGCTCCTTCC-3’ (IL1B forward) and 5’-AAGATGAAGGGAAAGAAGGTGC-3’ (IL1B reverse) at 65°C, *GAPDH* (5’-ACAGTCCATGCCATCACTGCC-3’ (GAPDH forward) and 5’-GCCTGCTTCACCACCTTCTTG-3’ (GAPDH reverse) at 70°C. Relative gene expression levels of each respective gene were calculated using the threshold cycle (2-ΔΔCT) method and normalized to *GAPDH* (28).

## Results

### Lactoferrin Binds to HAdV-C5, -D26 and -B35 and Increases Uptake by DCs

**Figure 1:**
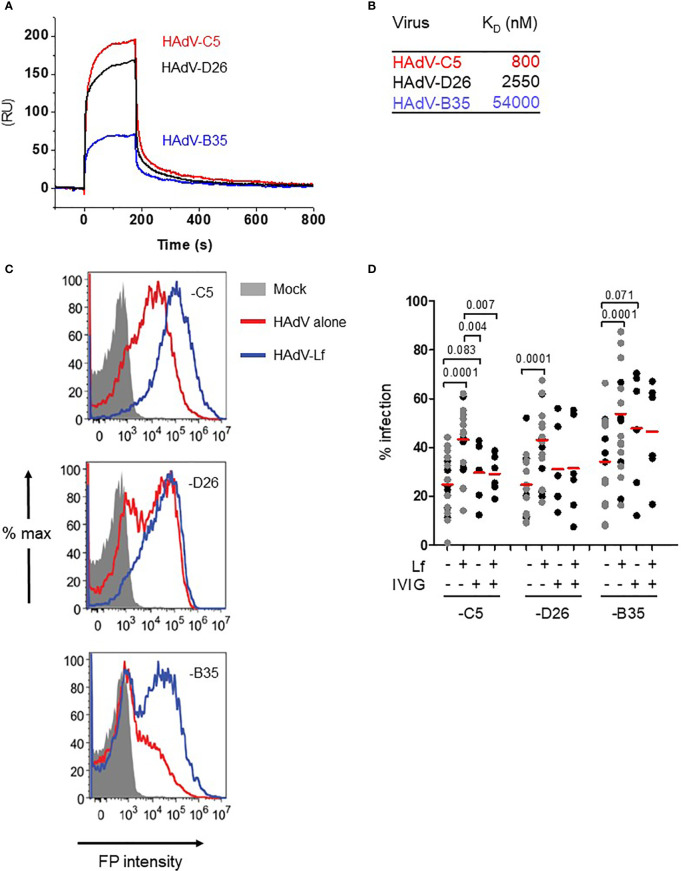
Lactoferrin binds to HAdVs and enhances uptake by DCs. **(A)** Representative sensorgrams of lactoferrin binding to HAdV capsids as assessed by surface plasmon resonance: HAdV-C5 (red), HAdV-D26 (black), and HAdV-B35 (blue) were covalently coupled to the CM5 sensor chip and lactoferrin was injected for binding comparison. For KD determination a range of 6.25 - 200 nM of lactoferrin was injected and the KD was calculated using a bivalent fitting model (RU = resonance units); **(B)** Relative affinity (KD) of lactoferrin for HAdV-C5, -D26 and -B35 capsids; **(C)** Representative flow cytometry profiles of DCs incubated with the HAdVs. DCs were mock-treated (grey) or incubated with HAdV-C5 (5,000 physical particles (pp)/cell), -D26 (20,000 pp/cell) or -B35 (1,000 pp/cell) (red) or with lactoferrin (blue). The samples were collected 24 h later, prepared for flow cytometry and 25,000 events were acquired/sample. FP = fluorescent protein (GFP or YFP); **(D)** Cumulative data from DCs using HAdVs complexed with lactoferrin, IVIG or both. Statistical analyses by two-tailed paired t-test for comparison of HAdV *vs.* HAdV + lactoferrin (n = 21). A subset of these samples (n ≥ 5, in black) was used for analyses between HAdV + lactoferrin vs. HAdV+ lactoferrin +IVIG.

At physiological pH, HAdV-C5, -D26 and -B35 have patches of negative charges in the hexon hypervariable regions that should potentiate cationic alarmin binding. We therefore quantified the affinity of human lactoferrin to each capsid by surface plasmon resonance (SPR). The HAdVs were immobilized on a CM5 sensor chip and then escalating doses of lactoferrin were injected over the sensor surfaces. We found that lactoferrin binds the three HAdVs with affinities (KD) that varied from 0.8 to 54 µM (**Figures 1A, B**) with a two-state association-dissociation reaction (**Figures S1A, B**).

In human myeloid and epithelial cells, HAdV-C5, -D26, and -B35 can use a range of receptors (29): HAdV-C5 predominantly uses CAR on epithelial cells and DC-SIGN (CD209) on DCs (30, 31); HAdV-D26 uses sialic acid-bearing glycans (32) and engage CD46 through a nonconventional interaction involving hexon (33); and HAdV-B35 predominantly uses CD46 (34). Of note, monocytes and DCs do not express detectable levels of CAR, all human cells have sialic acid-bearing glycans, and all nucleated human cells express CD46. We therefore tested the impact of lactoferrin on HAdV uptake using transgene expression from replication-defective vectors. Transgene expression was used as a surrogate assay for receptor engagement, internalization, cytoplasmic transport, docking at the nuclear pore, delivery of the genome to the nucleus, and transcription of the expression cassette. Because each HAdV type infects DCs with different efficacies, we chose a virus particle/cell ratio that generated approximately 30% of the cells expressing the transgene (see Material & Methods). Consistent with earlier reports (30), we found that lactoferrin increased (*p* ≤ 0.0001) HAdV-C5 uptake by human DCs (**Figures 1C, D**). HAdV-D26- and -B35-lactoferrin complexes were also taken up more efficiently (*p* ≤ 0.0001) than each HAdV alone (**Figures 1C, D**). Preincubating HAdVs with lactoferrin, adding lactoferrin to the cell medium before HAdV, or adding lactoferrin to the cell medium after the HAdVs, increased transgene expression (**Figure S1C**).

We previously showed that IVIG (pooled IgGs from >10,000 North American donors), which is rich in HAdV-C5 neutralizing antibodies, does not prevent infection of DCs by HAdV-C5 and, in some donors, induced greater uptake of HAdV-C5 (28, 35). To benchmark the effects induced by lactoferrin, we compared the HAdVs complexed with IVIG or lactoferrin. Globally, lactoferrin had a greater enhancing effect compared to IVIG (**Figure 1D**). As one of our long-term goals is to understand the innate response in a host with pre-existing HAdV immunity, we also combined lactoferrin and IVIG with each HAdV type. Our data demonstrate that IVIG abrogated of lactoferrin-enhanced uptake by HAdV-C5 and -D26 (**Figure 1D**). For the HAdV-B35-lactoferrin complex, the addition of IVIG induced a small decrease, which is consistent with the low seroprevalence of HAdV-B35 in North Americans (19). In addition to lactoferrin-enhanced uptake by DCs, we found that lactoferrin generally enhanced HAdV uptake by monocytes and monocyte-derived Langerhans cells (*p* < 0.03) (except HAdV-B35 in monocytes) (**Figures S1D, E**). These data demonstrate that, at physiological concentrations, lactoferrin binds to three HAdV types from different species and increased their uptake by phagocytes.

### HAdV-Lactoferrin Complexes Induce Cytokine Secretion

**Figure 2:**
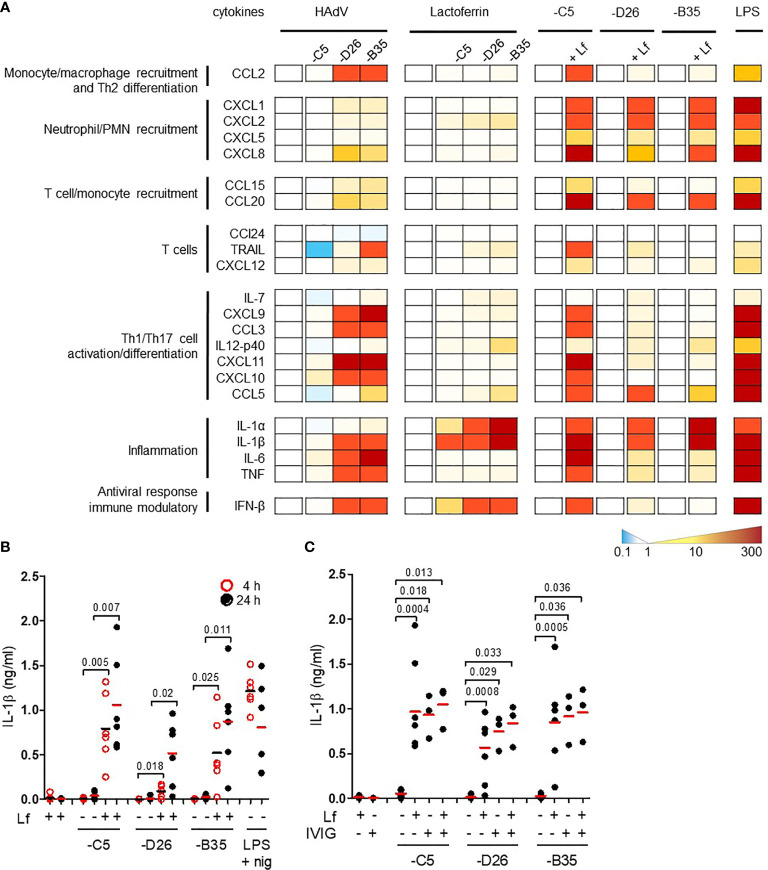
HAdV-lactoferrin complexes induce IL-1α and IL-1β. **(A)** DCs were incubated with HAdV-C5, -D26, or -B35 ± lactoferrin for 4 h and cytokine secretion in supernatants was assessed by Luminex. To the left of each set of columns is the baseline reference set at zero. For the 4 columns on the left (HAdV), the baseline is mock-infected cells; for the middle columns (Lactoferrin) the baseline reference is lactoferrin-treated cells; for the “paired” columns on the right (-C5, -D26, and -B35) the reference is HAdV-infected cells compared to HAdV-lactoferrin infected cells. Raw data can be found in **Figures S2A, B**. LPS was used as a control; **(B)** IL-1β release by DCs in the presence of HAdV-C5, -D26, and -B35 ± lactoferrin was assessed by ELISA at 4 (red circles) and 24 h (black dots) post-incubation (n = 6, statistical analyses by two-tailed paired t-test). As a control, cells were treated with LPS and nigericin; **(C)** DCs were incubated with HAdV complexed with lactoferrin, IVIG or lactoferrin + IVIG, for 4 h. IL-1β release was analysed 24 h post-incubation (n ≥ 3, statistical analyses by two-tailed paired t-test).

By influencing virus uptake, alarmins could induce DC maturation and an inflammatory response which would affect downstream adaptive responses. Conversely, virus-alarmins interactions could reduce the ability of an APC to present antigens, and therefore dampen an adaptive response. To determine whether HAdV-lactoferrin complexes influence DC maturation, we performed a pilot cytokine profile screen using a multiplex array. Because each component of the complexes influences DCs, it was necessary to provide individual blanked baselines to understand the impact of the complex. Compared to mock-treated DCs, HAdV-D26 and -B35 induced a greater cytokine response than HAdV-C5 (**Figure 2A**, the group of four columns on the left). Compared to lactoferrin-treated DCs, HAdV-lactoferrin complexes increased the release primarily in IL-1α and IL-1β (**Figure 2A**, the second group of four columns). When comparing the HAdV vs. the HAdV-lactoferrin complexes, the addition of lactoferrin induced a general increase in several cytokines (**Figure 2A**, the three pairs of columns on the right). The more pronounced effect of lactoferrin on HAdV-C5, may be due in part to the lower effect of HAdV-C5 alone on DCs (for raw data see **Figure S2A**) and/or the greater affinity lactoferrin for the HAdV-C5 capsid (**Figure 1**).

We then quantified the release of mature IL-1β in the supernatant. The addition of lactoferrin-HAdV complex induced greater IL-1β release than each virus alone, which further increased from 4 (*p* ≤ 0.025) to 24 h (*p* ≤ 0.02) post-stimulation (**Figure 2B**). Monocytes, which generate relatively low levels of IL-1β, also released more when challenged with HAdV-D26-lactoferrin (*p* < 0.023) and HAdV-B35-lactoferrin (*p* < 0.042) compared with the HAdV alone, respectively (**Figure S2B**).

To benchmark the impact of lactoferrin, we compared it to the effect induced by IVIG. We found that IVIG and lactoferrin were similar in their ability to induce IL-1β release, and that when combined a trend toward an additive effect was apparent, suggesting a link to increased particle uptake (**Figure 2C**). Using a phenotypic (CD86 surface level) and functional (changes in phagocytic capacity) assays, we found that lactoferrin increased DC maturation (**Figures S2C, D**). Together, these data demonstrate that lactoferrin, a pleiotropic HDP, affects the innate immune response to HAdV-C5, -D26, and -B35.

### TLR4 is Involved in HAdV-Lactoferrin Induced DC Maturation

**Figure 3:**
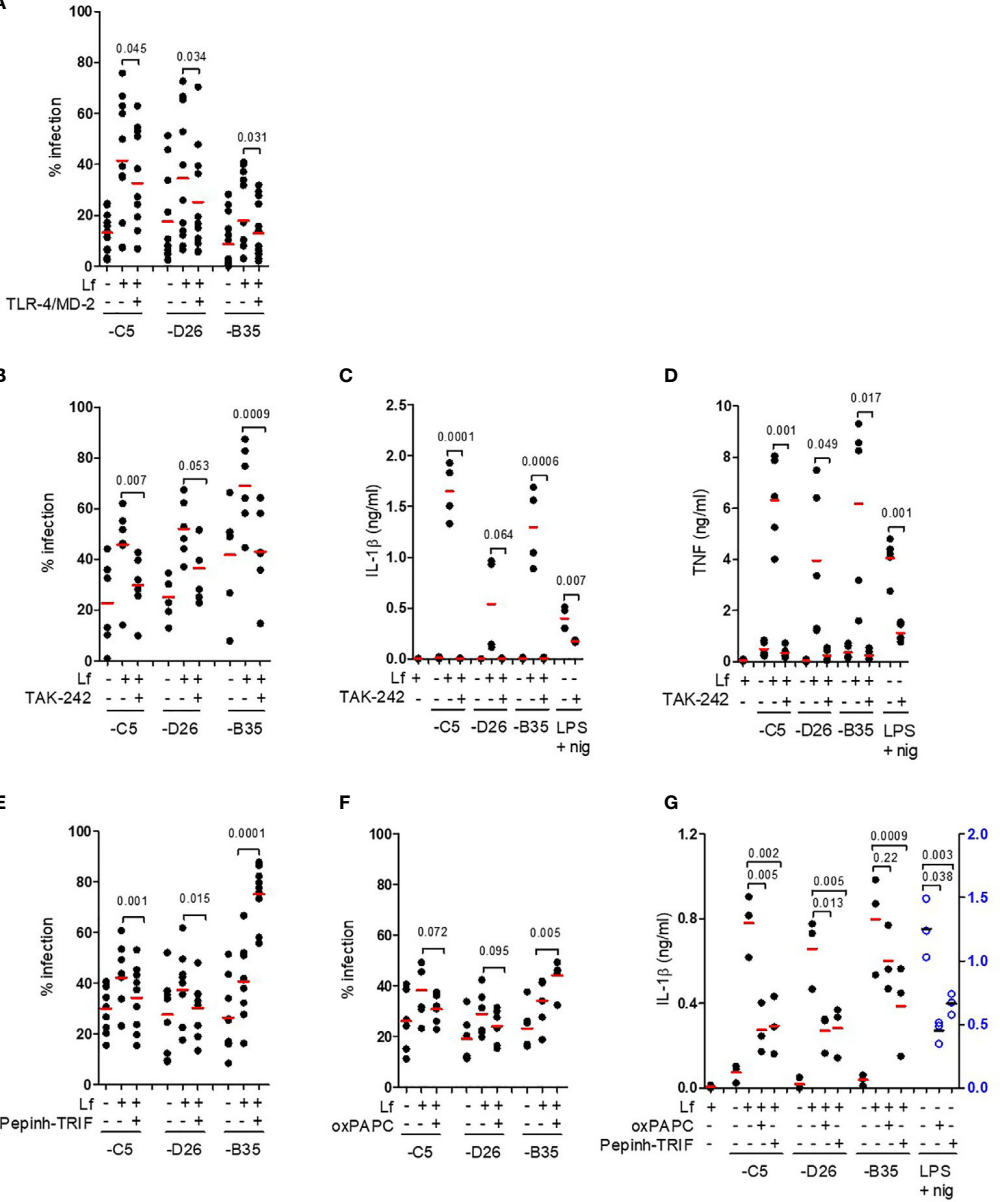
TLR4 engagement and signalling involved in HAdV-lactoferrin DC uptake and maturation. **(A)** HAdV-lactoferrin complexes were incubated with recombinant TLR4/MD-2 for 30 min. and then added to DCs. Uptake was quantified 24 h post-incubation by flow cytometry (n =11, statistical analyses by two-tailed paired t-test); **(B)** DCs were pre-treated for 1 h with TAK-242, HAdV-lactoferrin complex uptake was analysed 24 h post-incubation by flow cytometry (n = 6, statistical analyses by two-tailed paired t-test); **(C)** IL-1β release following pre-treatment with TAK-242 (n ≥ 3 statistical analyses by t-test); **(D)** TNF levels 24 h post-incubation ± TAK-242 (n ≥ 3, statistical analyses by two-tailed paired t-test); **(E)** Percent infection following inhibition with Pepinh-TRIF (n ≥ 6, statistical analyses by two-tailed paired t-test); **(F)** Percent infection following inhibition with oxPAPC (n ≥ 6, statistical analyses by two-tailed paired t-test); **(G)** IL-1β release from DCs incubated with HAdV-lactoferrin ± oxPAPC and Pepinh-TRIF (n = 3, statistical analyses by two-tailed paired t-test).

We then hypothesized that lactoferrin-enhanced HAdV uptake was due to alternative receptor engagement because lactoferrin alone can increase DC maturation by interacting with TLR4 (5, 36–38). In some myeloid cells, TLR4 forms a complex with MD-2 for ligand binding (39), and with CD14 for internalization (40). MD-2 acts as a co-receptor for recognition of exogenous and endogenous ligands (39, 41, 42). In addition, Doronin et al. proposed that HAdV-C5 interacts with murine TLR4 *via* a murine coagulation FX-bridge (23). Yet, human FX did not act as a bridge for HAdV-C5 *via* TLR4 on human DCs (24). To determine whether HAdV-lactoferrin complexes engage TLR4 on the cell surface, we incubated the HAdV-C5, -D26 and -B35 lactoferrin complexes with recombinant TLR4, MD-2, or TLR4/MD-2 dimers, or blocked CD14 on the cell surface with an anti-CD14 antibody. When using these protein-based assays, we found a reduction (*p* ≤ 0.045) in the uptake of HAd-C5, -D26 and -B35 in the presence of the TL4/MD-2 dimer (**Figure 3A** **).** Recombinant TLR4 alone had no notable impact, the anti-CD14 antibody suggested a mild trend toward (*p* ~ 0.1) inhibition of uptake, whereas recombinant MD-2 inhibited (*p* ≤ 0.045) only HAdV-D26 (**Figure S3A**).

To address the possible involvement of the cytoplasmic TLR4 tail, we used a cell-permeable cyclohexene-carboxylate (TAK-242), to disrupt interactions with the adaptor molecules TIRAP and TRAM (43–45). We found that TAK-242 reduced (*p* ≤ 0.007) lactoferrin-enhanced uptake of HAdV-C5 and -B35, while its impact on -D26 was less prominent (*p* ≤ 0.053) (**Figure 3B**). The impact of TAK-242 on IL-1β release (**Figure 3C** **)** mirrored the uptake assay: we found a reduction (*p* ≤ 0.021) following incubation with HAdV-C5 and -B35 lactoferrin challenge, but less prominent (at least statistically) for -D26 (*p* > 0.05). Importantly though, in each case pre-incubation with TAK-242 reduced IL-1β levels to near background. As expected, TAK-242 also reduced (*p* ≤ 0.05) TNF secretion (**Figure 3D**). Of note, the disruption of TLR4-TIRAP/TRAM interactions by TAK-242 did not impact lactoferrin-enhanced uptake by monocytes or Langerhans cells (**Figures S3B, C**), suggesting differences in TLR4 function between these cell types. We then addressed the role of the TLR4 complex using Pepinh-TRIF to prevent cytoplasmic TLR4 - TRIF interactions. We found that Pepinh-TRIF decreased (*p* < 0.015) uptake of HAdV-C5- and -D26-lactoferrin and, surprisingly, increased (*p* < 0.001) lactoferrin-HAdV-B35 uptake (**Figure 3E**).

To address the involvement of the extracellular TLR4 domain, we used oxPAPC to prevent TLR4 - MD2 interactions. While oxPAPC did not affect uptake of HAdV-C5- or HAdV-D26-lactoferrin complexes, oxPAPC increased (*p* < 0.005) HAdV-B35-lactoferrin uptake (**Figure 3F**). The impact of oxPAPC and Pepinh-TRIF on IL-1β release was more consistent and led to notable decrease (*p* ≤ 0.013), except for oxPAPC on HAdV-B35 (*p* > 0.2) (**Figure 3G**). Of note, lactoferrin, TAK-242, and Pepinh-TRIF did not change TLR4 surface levels at most concentrations (**Figure S3D–F**), whereas oxPAPC increased TLR4 levels at the concentrations used in this study (**Figure S3G**).

In addition, lactoferrin is post-translationally cleaved to generate lactoferricin, a biologically active N-terminal fragment of 49 aa. Lactoferricin also binds to negatively charged hexon hypervariable regions (HVRs) of HAdV-C5, -A31 and -B35 (46). To determine whether lactoferricin could mimic the effects of lactoferrin, we incubated the former with the HAdV vectors. We found no notable increase in HAdV uptake or IL-1β release (**Figures S3H, I**), suggesting that the C-terminus of lactoferrin influences HAdV-TLR4 interactions. Together, these data demonstrate that interfering with TLR4 engagement/signalling reduces HAdV-lactoferrin-mediated transgene expression and DC maturation in the case of HAdV-C5 and -D26.

### HAdV-Lactoferrin Complexes Induce NLRP3 Inflammasome Formation

**Figure 4:**
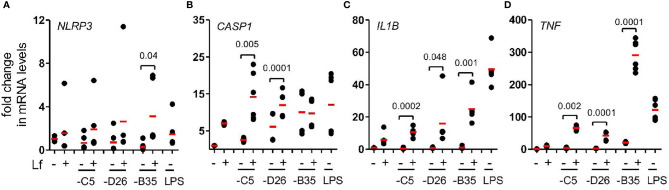
HAdV-lactoferrin complexes induce IL-1β *via* an NLRP3 inflammasome mRNA levels of inflammasome components were analysed using qRT-PCR 4 h post-incubation. Total RNA was isolated from DCs and cDNA samples were analysed in triplicate. The GAPDH mRNA was used as an internal standard. **(A)** *NLRP3*; **(B)** *CASP1*, **(C)** (*IL1B* and **(D)** *TNF*; (n ≥ 3, statistical analyses were by two-tailed paired t-test).

The inflammasome is a multiprotein cytosolic platform consisting of a PRR that induces nucleation of ASC (apoptosis-associated speck-like protein containing a CARD) and recruitment of pro-caspase 1. Pro-caspase-1 auto-activation can be followed by removal of the N-terminus of gasdermin D (GSDMD), which initiates the loss of plasma membrane integrity *via* pore formation (47). Classic NLRP3 inflammasome formation (canonical and non-canonical) is preceded by transcriptional priming event (signal 1) needed to produce inflammasome components and cytokines (48). In human mononuclear phagocytes, TLR4 engagement by LPS can also induce an alternative NLRP3 inflammasome activation that does not need transcriptional priming (22). To determine whether HAdV-lactoferrin engagement of TLR4 induced transcription of inflammasome components, we used RT-qPCR to examine selective mRNAs. We found that in most cases HAdV-lactoferrin complexes increased *NLRP3*, *CASP1*, *IL1B*, and *TNF* mRNAs compared to the HAdVs alone (**Figures 4A–D**).

**Figure 5:**
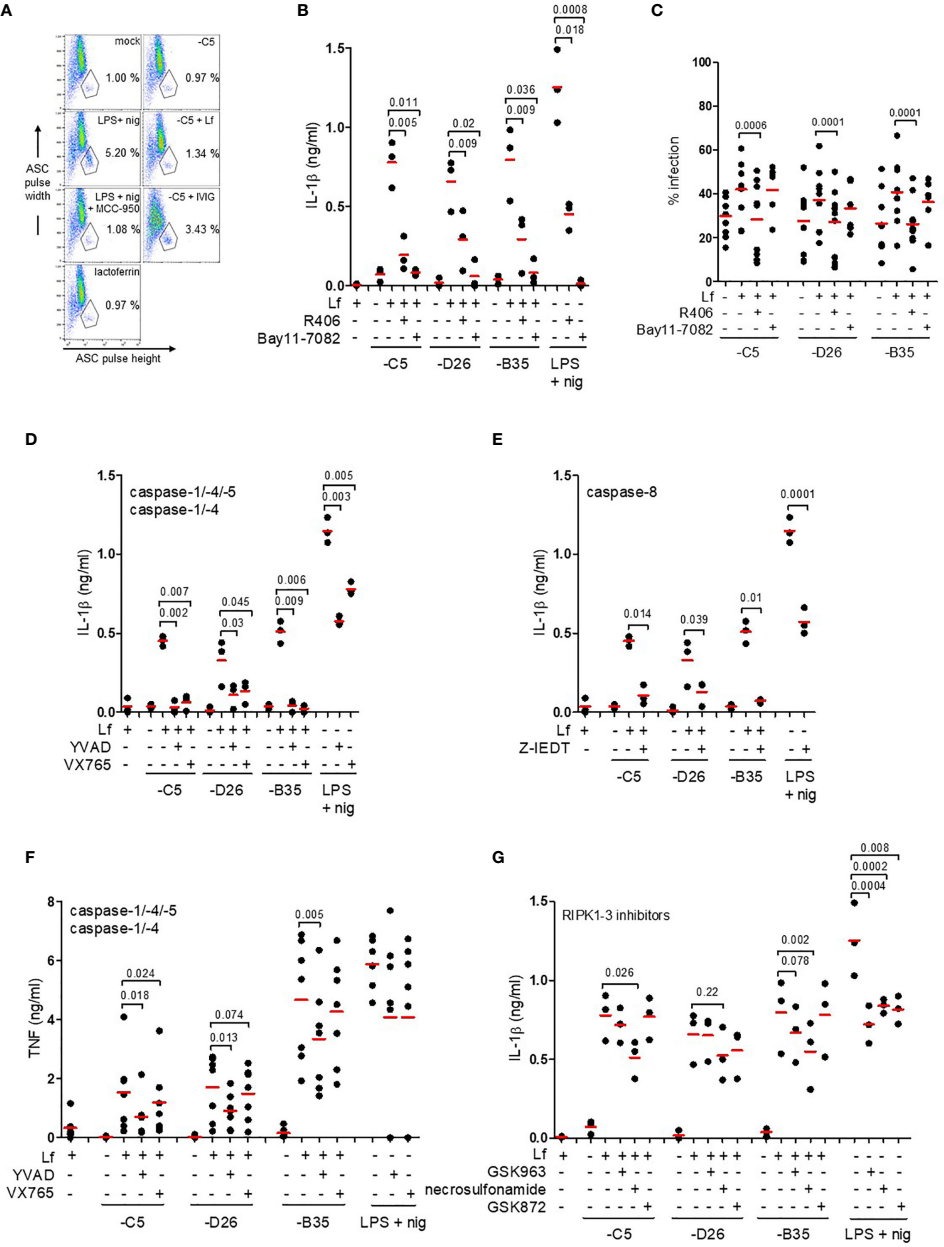
IL-1β release *via* an NLRP3 inflammasome and caspase-1. **(A)** Flow cytometry-based assay for ASC aggregation (pyroptosome formation): inflammasome formation was induced by incubating DCs with HAdV-C5-lactoferrin complexes for 3 h. LPS + nigericin and HAdV-C5-IVIG complexes were used as positive controls to identify inflammasome-positive cells. MCC-950 was used to inhibit NLRP3 inflammasomes. Lactoferrin was used as a negative control. Cells were stained with anti-ASC antibodies. Inflammasome-positive cells were identified as ASC-width low and ASC-height high; **(B)** IL-1β release in response to HAdV-lactoferrin complexes: DCs were pre-treated with R406 and Bay11-7082. LPS/nigericin was used as a positive control (n ≥ 3, statistical analyses by two-tailed paired t-test). **(C)** The percentage of infection of DCs by HAdV-lactoferrin complexes after pre-incubation of DCs with R406 and Bay11-7082. **(D)** Impact of caspase-1, -4, and -5 inhibition on IL-1β release. **(E)** Impact of caspase-8 inhibition on IL-1β release. **(F)** TNF release in response to HAdV-lactoferrin complexes in DCs pre-treated with YVAD or VX765. **(G)** IL-1β release in response to HAdV-lactoferrin complexes in DCs pre-treated with RIPK1-RIPK3 pathway inhibitors GSK963, necrosulfonamide or GSK872. N ≥ 3 in all assays. Statistical analyses by two-tailed paired t-test.

During inflammasome formation, ASC changes from being distributed throughout the cytoplasm to an aggregate of ~1 µm diameter upon nucleation by NLRP3. ASC nucleation can be directly visualized by changes in the fluorescence pulse width ratio. While this flow cytometry-based assay does not allow quantification of all the cells that had, or will contain, an inflammasome, it does provide a semi-quantitative snapshot of inflammasome formation at a given time. In our hands, IVIG does not contain detectable levels of HAdV-D26 or -B35 neutralizing antibodies, while the titres of HAdV-C5 neutralizing antibodies are high and promote inflammasome formation in DCs. Therefore IgG-complexed HAdV-C5 was used as a benchmark. We found that ~1% of mock-treated DCs contained an inflammasome. LPS/nigericin and HAdV-C5-IVIG induced 5.2 and 3.4% inflammasome-positive cells, respectively [~40% of the DCs will undergo pyroptosis in 8 h when incubated with this concentration of HAdV-C5-IVIG (28)]. We found a ~25% increase in the number of inflammasome-positive DCs 3 h post-challenge with HAdV-C5-lactoferrin complexes (**Figure 5A**), consistent with increased IL-1β release following challenge with HAdV-C5-lactoferrin and suggestive of inflammasome formation.

IL-1β release is associated with both classic and alternative activation of NLRP3 inflammasomes. To try to identify the HAdV-lactoferrin-associated trigger(s), DCs were treated with KCl (to prevent K^+^ efflux), NAC (a reactive oxygen species scavenger), or MDL (a cathepsin B inhibitor) (47). In our hands, the addition of extracellular K^+^, NAC or MDL did not (*p* > 0.07) modify the release of IL-1β or lactoferrin-enhanced uptake (**Figures S4A, B**). When DCs were pre-incubated with R406 (which blocks MyD88-Syk interactions and interrupts the signalling between TLR4 engagement and NLRP3 inflammasome regulation), or Bay11-7082 [an NLRP3 inhibitor (49)], IL-1β release was reduced (*p* ≤ 0.036) in response to HAdV-lactoferrin complexes (**Figure 5B**). While Bay11-7082 had no impact on lactoferrin-enhanced uptake, R406 reduced uptake of all HAdVs (**Figure 5C**), suggesting that MyD88-Syk signalling was necessary to enhance HAdV uptake.

We then inhibited the caspases that are involved in pyroptosis using Z-YVAD-FMK (caspase-1, -4 and -5), VX765 (caspase-1 and -4) or Z-IETD (capsase-8). Globally, all the inhibitors reduced IL-1β release to some extent (**Figures 5D, E**). In contrast to VX765 and Z-IETD, Z-YVAD-FMK also reduced (*p* ≤ 0.018) lactoferrin-associated TNF secretion (**Figure 5F** and **Figure S4C**). The caspase inhibitors had no consistent effect on lactoferrin-enhanced uptake of the HAdVs (**Figure S4D**). In addition to the direct effects of TLR4 engagement and signalling, it was possible that TNF secretion induced an autocrine response and inflammasome activation *via* a RIPK1-RIPK3-caspase-8 pathway (50). Inhibition of the TNFR signalling using GSK963, necrosulfonamide, and GSK872 had no significant effect on uptake of the HAdVs (**Figure S4E**). By contrast, IL-1β release was reduced (*p* ≤ 0.026) by necrosulfonamide during lactoferrin-enhanced uptake of HAdV-C5 and -B35 **(** **Figure 5G**). For combined analyses broken down by HAdV type see **Figure S4F–H**. Together, these data demonstrate that HAdV-lactoferrin complexes induced NLRP3 inflammasome formation and IL-1β release *via* the activation of the TLR4 pathway. Additionally, an autocrine effect of TNF receptor may influence IL-1β release.

### IL-1β Release Without the Loss of Membrane Integrity

**Figure 6:**
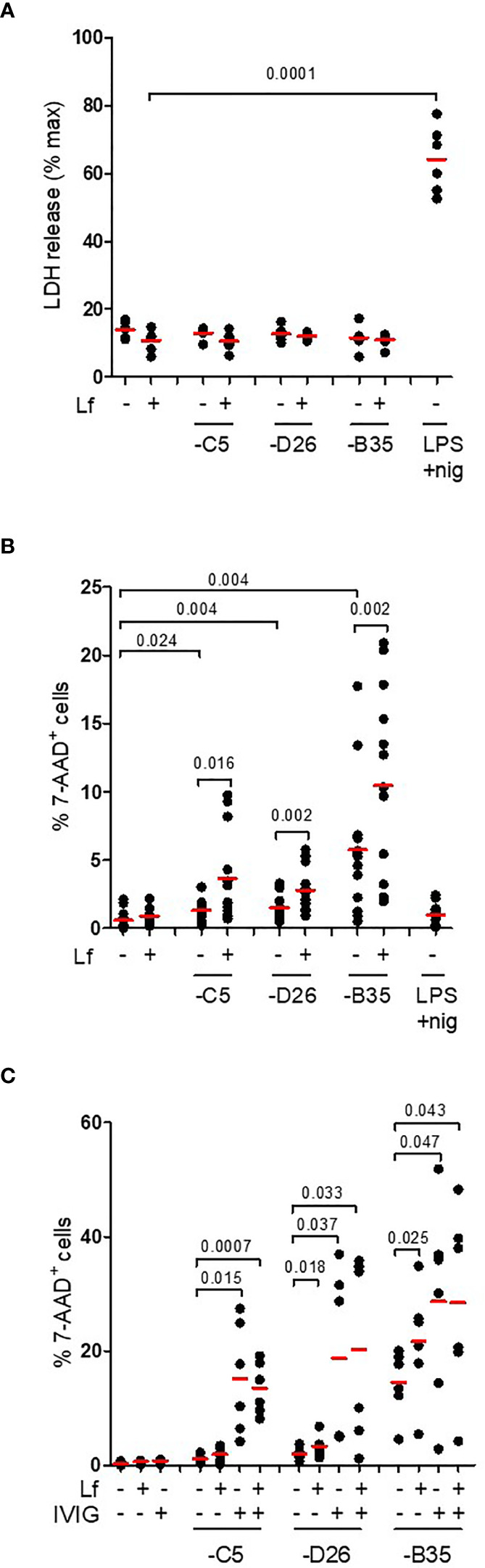
IL-1β release without loss of membrane integrity. **(A)** DCs were challenged with HAdV-lactoferrin complexes, HAdV alone, or LPS/nigericin. Loss of cytosolic content was quantified by LDH activity in the supernatant at 4 h post-incubation (n = 6); **(B)** Plasma membrane integrity, analysed 24 h post-incubation using 7-AAD uptake, was quantified using flow cytometry (n = 13); **(C)** DCs were incubated with HAdV complexed with lactoferrin, IVIG or lactoferrin + IVIG. Plasma membrane integrity was analysed by 7-AAD uptake by flow cytometry at 24 h post-incubation (n ≥ 3). Statistical analyses by two-tailed paired t-test.

In contrast to classic NLRP3 inflammasome activation, the alternative pathway does not induce complete loss of cell membrane integrity (as based on L-lactate dehydrogenase (LDH) release) (22). This is thought to be due to ESCRT III pathway repairing pores in the plasma membrane induced by limited levels of GSDMD cleavage (51). To determine if HAdV-lactoferrin complexes induce pore formation and the release of large intracellular proteins, we quantified extracellular levels of LDH activity at 4 h postinfection and found no increase (*p* > 0.5) in any conditions (**Figure 6A**). To determine whether HAdV-lactoferrin complexes were able to have a long-term impact on DC membrane integrity, we added a fluorescent marker of viability (7-AAD) to the DCs and quantified fluorescent intensity by flow cytometry. At 24 h post-challenge, the percentage of 7-AAD^+^ cells induced by HAdV-lactoferrin complexes was greater (*p* ≤ 0.024) than lactoferrin- or HAdV-challenged cells (**Figure 6B**). Moreover, when lactoferrin was added to HAdV–IVIG complexes, we found a trend toward a further increase in the percentage of 7-AAD^+^ DCs (**Figure 6C**). We concluded that while membrane integrity may be perturbed and allow uptake of small molecules, within the time frame of our assays cytosolic proteins are not released into the medium and therefore pyroptosis is not efficiently engaged or aborted.

## Discussion

Deconstructionist approaches using binary systems to understand HAdV receptor engagement, trafficking, and immunogenicity provide a foundation to understand virus-cell interactions. Using cell lines, which allow reproducibility but generate inherently biased results, also limits the clinical relevance that one would generate by using primary cells from a random cohort. Combinatorial assays using human blood components, in particular phagocytes, can generate insight into clinically relevant HAdV issues such as the induction of an innate immune response. Here, we show how an alarmin influences the response of human DCs to three HAdV types. We examined pathways from receptor engagement, signalling, transcription, inflammasome formation/activation and cytokine release.

Throughout our study HAdV-C5 and -D26 tended to have similar profiles in most assays, whereas -B35 was notably different. These differences may be attributed to the relative affinity of lactoferrin to the capsid (C5 > D26 > B35), or interaction with their respective panoply of receptors. In addition, work in T cells that shows CD46 primes the NLRP3 inflammasome and therefore a possible binary engagement through TLR4 and CD46 could impact the response to HAdV-B35-lactoferrin complexes (52). The breadth of the lactoferrin-enhanced uptake of the three HAdVs suggest that the interactions are charge based because of the significant differences in the HVR sequences, which make up much of the surface area of HAdVs. Previous studies demonstrated that some HDPs attenuate HAdV infection of epithelial-like cells (8, 26, 53–56). By contrast, our results are similar to Adams et al. (9) and demonstrate that lactoferrin enhances HAdV uptake by human monocytes, DCs, and Langerhans cells. By delving deeper into these initial observations, our study sheds light onto the mechanisms by which an HDP connects HAdV infection of mucosal tissues, or during vaccination, to drive innate and adaptive immune responses.

Mechanistically, it appears that lactoferrin reduces uptake of targeted cells and increases uptake into phagocytes, which provokes a pro-inflammatory and antiviral cytokine response. In combination with vaccination studies, our study provides insight into how lactoferrin modifies adaptive immune responses against HAdV vectors and/or the transgene. What do our results mean for HAdV-based vaccines? We posit that lactoferrin acts like a natural adjuvant and likely increases an anti-viral response. Should lactoferrin be included as an “adjuvant” in HAdV-based vaccines? Or, is there an excess of lactoferrin rapidly produced following vector injection? Could HAdV-lactoferrin formulation significantly reduce the dose needed for an optimal response to vaccines? Or conversely, does the lactoferrin-mediated uptake by phagocytes limit the efficacy of HAdV-based vaccines by precluding infection of targeted cells and long-term, steady expression of the transgene (which theoretically is ideal for broad and long-term responses)? Following the rare and severe thromboses and thrombocytopenia linked with HAdV-D26 and the chimpanzee AdV (ChAdOx1) based vaccines, the need to understand the human innate immune response is even greater.

Classical NLRP3 inflammasome activation involves a two-step process (57): PRR-derived signal to upregulate transcription of inflammasome components and NLRP3 posttranslational modification. Following engagement of TLR4, its TIR domain recruits MyD88 and TIRAP, which bridge TLRs to IRAK and MAPK family members that activate NF-κB, AP-1, and IRF. These latter pathways initiate transcription of genes coding for inflammasome components and proinflammatory cytokines (58, 59). The TIR domain also recruits TRAM and TRIF to activate the kinases TBK1 and IKKϵ to promote type I IFN expression (43). Together, these pathways prime an adaptive antiviral response. NLRP3 then detects perturbations of cellular integrity associated with K^+^ efflux (signal 2). Consequently, Nek7–NLRP3 interaction leads to pyroptosome assembly and caspase-1-induced maturation of pro-IL-1β and pro-GSDMD. The alternative pathway consists of NLRP3-ASC-pro-caspase-1 signalling and IL-1β release without the loss of cytoplasmic content. Yet, the alternative pathway delineated in this study is not an indisputable fit and likely reflects the variability between LPS and HAdVs. TLR4-mediated endocytosis, which is well characterized for LPS, depends on the homodimerization of TLR4. LPS, the quintessential TLR4 ligand, is extracted from gram negative bacteria by CD14, which then transfer it to MD-2, which interacts directly with TLR4. TLR4 dimerization is induced by the lipid A region of LPS. Given the icosahedral shape and the size (~90 nm) of the HAdV-lactoferrin complex, one would expect that lactoferrin binds to multiple sites on the capsid and induces TLR4 dimerization directly, or possibly *via* assemblage of multiple dimers. Dimerization is then associated with CD14-dependent migration (60) to cholesterol-rich regions of the plasma membrane and endocytosis *via* a TLR4 ectodomain-dependent mechanism. While this picture appears partially consistent with the uptake of HAdV particles, CD14 levels on monocyte-derived DCs are low or absent, suggesting that migration to lipid rafts is *via* a different pathway.

Most TLR4 agonists do not have complex intracellular processing. This is not the case for HAdVs. The endosomolytic activity of protein VI, an internal capsid protein, prevents the complete degradation of IgG-complexed HAdVs in DCs by enabling the escape of the partially degraded HAdV capsid from endocytic vesicles/lysosomes into the cytoplasm (61, 62). However, this then causes the HAdV double-stranded DNA genome to become accessible to AIM2 (absent in melanoma 2) and in turn to initiate inflammasome formation. The makeup and processing of TLR4-associated vs. Fc receptor-associated endocytic vesicles is, to the best of our knowledge, unknown. From the data presented here, it appears that TLR4-associated endocytic vesicles engage the inception of an NLRP3 inflammasome. Of note, we did not detect an involvement of the cGAS pathway (the inhibitor RU.512 had no effect on HAdV-mediated transgene expression or IL-1β release, data not shown) suggesting that in our assays TLR4-mediated endocytosis was not associated with significant degradation of the HAdV capsid. These data are also consistent with increased transgene expression from the replication-defective vectors. Caspase 1 cleavage of GSDMD abolishes its intra-molecular auto-inhibition and induces pore-like structures of ~15 nm in diameter in the plasma membrane to breakdown the ion gradients. Alternative inflammasome activation can be triggered by a unique signal. LPS sensing induces a TLR4-TRIF-RIPK1-FADD-CASP8 signalling axis, resulting in activation of NLRP3 by cleavage of an unknown caspase-8 substrate independent of K^+^ efflux. The alternative NLRP3 complex likely has a modified stoichiometry. Although caspase 1 becomes mature and cleaves pro-IL-1β, pyroptosis is not induced and IL-1β release by an unconventional mechanism that functions independently of GSDMD. Why HAdV-lactoferrin-challenged cells do not release a significant amount of cytoplasmic content may be due to the spatial and temporal signals cells are receiving during the activation phase. In classic NLRP3 inflammasome activation, signal 1 is received well before signal 2 (NLRP3 engagement). In our assays, HAdV-lactoferrin induced signals are received immediately before the NLRP3 induction. Not surprisingly, inhibition of the NF-κB pathway decreased IL-1β levels. The poor coordination of transcriptional priming and de-ubiquitination of NLRP3 (63) may preclude pyroptosis, and favour an immune response with a longer duration and trafficking of DCs to lymph node to induce an adaptive immune responses. Inflammasome activation is thought to be crucial for the induction of cellular and humoral immune responses in the context of vaccinations. The involvement of the HAdV-lactoferrin NLRP3 axis may help drive the T-cell response toward a Th1 phenotype. Importantly, controlling an excessive inflammatory response is necessary. In addition to the expression of IL-1β, we also found notable levels of IL-1α. It is also possible that the effects of IL-1α supersede or preclude pyroptosis because IL-1α promotes the expression of genes involved in cell survival (64).

Finally, our results may also resolve one of many conundrums associated with the differences between murine and human responses to HAdVs. If murine HDPs also interact HAdVs and induce a TLR4-associated pro-inflammatory response in the mouse liver (23), then this complex partially resolves the mouse-man paradox. Whether HAdV-coagulation factor-HDP complexes are produced following intravenous injection in mice has not been addressed.

**Figure 7:**
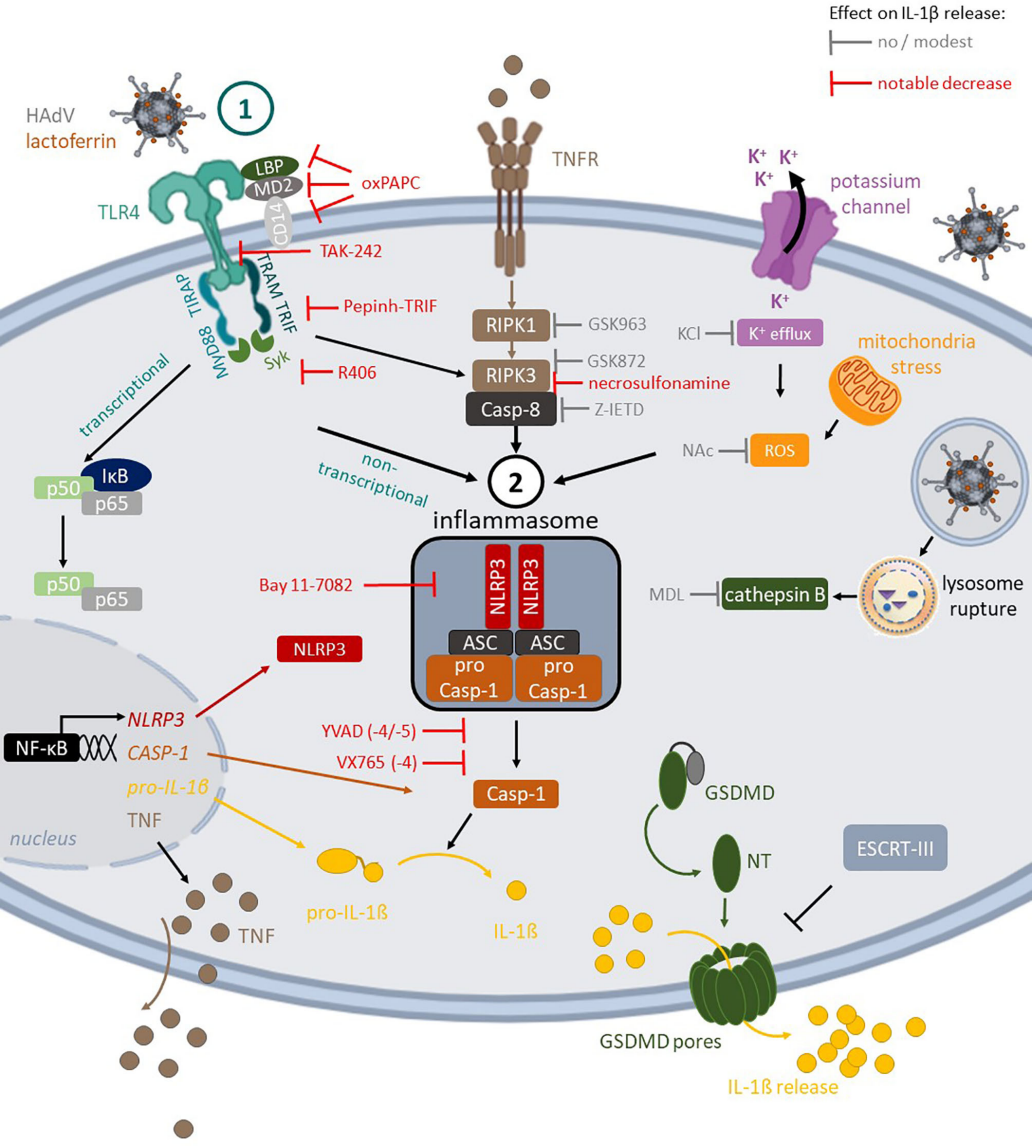
TLR4-mediated HAdV-lactoferrin uptake in DCs and IL-1β release Lactoferrin binds to HAdV capsid and retargets the capsid toward TLR4 complex on the cells surface. Following TLR4 engagement, its TIR domain recruits MyD88 and TIRAP, which bridge TLRs to IRAK and MAPK family members that activate NF-kB, AP-1, and IRF. This priming event initiates transcription of genes coding for inflammasome components (e.g., NLRP3 and IL-1β). Under prototypic conditions, DCs detect a second perturbation (signal 2) that induced ROS release (mitochondrial stress) or K^+^ efflux (perturbations of cellular integrity), and/or cathepsin B release from lysosome rupture. These pathways do not appear to be activated by HAdV-lactoferrin complexes. In addition to TLR4 pathways, the RIPK1-RIPK3 pathway is activated through an autocrine-TNF release. During inflammasome formation, pro-caspase-1 auto-activation induces cleavage of pro-IL-1β and likely GSDMD, which will initiate, but not complete, the loss of plasma membrane integrity *via* pore formation, allowing IL-1β release. Twenty-four hours post-challenge, DC membrane integrity is intact, consistent with the involvement of ESCRT-III complex and repairing GSDMD pores.

In conclusion, using combinatorial assays and primary human blood cells we detailed the multifaceted interactions between three HAdVs, a DAMP (lactoferrin), and PRRs (TLR4 & NLRP3) at the interface of innate and adaptive immunity in humans **(** **Figure 7**). These data directly address how the multiple layers of the innate and adaptive immune responses coordinate reactions to pathogens and HAdV-based vaccines.

## Data Availability Statement

The datasets presented in this study can be found in online repositories. The names of the repository/repositories and accession number(s) can be found in the article/**Supplementary Material**.

## Author Contributions

Study design & conception: KE and EK. Project direction: EK. Performed experiments; CC, KE, THT, TPT, OP, CH, and JB. Analysed data: all authors. Wrote the manuscript: CC, KE, THT, and EK. Secured funding: EK. Data and materials availability: all materials can be obtained through an MTA. All authors contributed to the article and approved the submitted version.

## Funding

This work benefited from support by the LabEx EpiGenMed, an “Investissements d’avenir” program, Université de Montpellier (EK), Ph.D. fellowship from the Vietnamese Minister of Education (TPT), Ph.D. fellowship from the French Minister of Education (OP), the Innovative Medicine Initiative EboVac2 (#115861) (EK), TransVac2 (EC Horizon 2020 #730964) (EK), and the Institut de Génétique Moléculaire de Montpellier (EK). The funders had no role in study design, data collection and analysis, decision to publish, or preparation of the manuscript.

## Conflict of Interest

The authors declare that the research was conducted in the absence of any commercial or financial relationships that could be construed as a potential conflict of interest.

## Supplementary Material

Supplementary Figure 1 Analyses of lactoferrin binding to HAdV-C5, -D26 and -B35 by SPR **(A)** HAdV-C5, -D26, and -B35 were covalently coupled to a CM5 sensor chip and escalating doses of lactoferrin (6.25 - 200 nM) for KD determination. Depicted are overlaid sensorgrams (RU = resonance units); **(B)** The association and dissociation rates of lactoferrin for HAdV-C5, -D26 and -B35 capsids **(C)** Representative flow cytometry profiles of cells incubated with HAdVs ± lactoferrin. DCs were mock-treated (grey), incubated with HAdV -C5, -D26 and -B35 alone (red), with lactoferrin complexed with HAdV (blue), with HAdV for 30 min and then lactoferrin (green) or with lactoferrin for 30 min and then HAdV (purple). Fluorescence was analysed 24 h post-incubation; **(D)** monocytes and **(E)** LCs were incubated with HAdVs ± lactoferrin and fluorescence was analysed 24 h post-incubation (n = 5, statistical analyses by two-tailed paired t-test).

Supplementary Figure 2 HAdV-lactoferrin induced cytokine secretion **(A)** Raw data of Luminex assay of DCs ± HAdVs ± lactoferrin, plus controls. Data indicated total protein level. **(B)** Freshly isolate human CD14^+^ monocytes were incubated with HAdV-C5-, -D26, or -B35 ± lactoferrin. The supernatants (24 postintubation) were used to quantify IL-1β release (n ≥ 3, statistical analyses by two-tailed paired t-test); **(C, D)** DCs were incubated with HAdV-lactoferrin complexes for 24 h. Phenotypic maturation was assessed by CD86 surface levels expression and functionally **(D)** by fluorescent dextran uptake. Cells were incubated at 4°C or 37°C for 30 min with 1 mg/ml TRITC-labelled dextran, washed with PBS, fixed with 4% PFA and analysed by flow cytometry (lower fluorescence = lower phagocytosis = greater maturation) (n ≥ 3, statistical analyses by two-tailed paired t-test).

Supplementary Figure 3 TLR4 inhibitors reduce infection of HAdV-lactoferrin complexes **(A)** HAdV-lactoferrin complexes were incubated with recombinant TLR4, TLR4/MD-2, MD-2 proteins for 30 min, or DCs were pre-incubated with anti-CD14 antibody for 30 min. Then, HAdV-lactoferrin-recombinant protein complexes were added to DCs or HAdV-lactoferrin were added to treated DCs. Infection was analysed 24 h post-incubation by flow cytometry (n = 5, statistical analyses by two-tailed paired t-test); **(B, C)** Monocytes and LCs were treated for 1 h pre-infection with 1 µg/ml of TAK-242. HAdV-lactoferrin complex infection was analysed 24 h post-incubation by flow cytometry (n ≥ 5, statistical analyses by two-tailed paired t-test); **(D-G)** TLR4 surface levels were analysed at 24 h after DC treatment with decreasing concentrations of lactoferrin (0.9 - 7 µg/ml), TAK-242 (25 - 200 µg/ml), Pepinh-TRIF (6.25 - 50 µg/ml) or oxPAPC (7.5 - 60 µg/ml). The assays were performed in triplicate with cells from a single donor and the levels represents the average. The R^2^ score correspond to the coefficient of determination for linear regression. **(H, I)** The HAdVs were complexed with lactoferricin (a gift from H. Jenssen, Roskilde Universitet) and DC infection and IL-1β release were analysed at 24 h post-incubation, respectively (n = 4, statistical analyses by two-tailed paired t-test).

Supplementary Figure 4 Effect of pharmacological inhibition on HAdV infection **(A)** IL-1β release following inhibition of pyroptosis initiators (n ≥ 5, statistical analyses by two-tailed paired t-test) **(B)** The percentage of infection of DCs pre-treated with inhibitors of pyroptosis initiators (n = 3, statistical analyses by two-tailed paired t-test); **(C)** TNF secretion by DC pre-treated with caspase 8 inhibitors; DCs were treat with Z-IETD for 1 h. Cells were incubated with HAdV-lactoferrin complexes for 24 h and the supernatant was collected for TNF quantification (n ≥ 5, statistical analyses by two-tailed paired t-test); **(D)** The percentage of infection of DCs pre-treated with caspase 1, 4, 5, 8 inhibitors (n ≥ 6, statistical analyses by two-tailed paired t-test), **(E)** The percentage of infection of DCs pre-treated with RIPK1 inhibitor GSK963 and RIPK3 inhibitors necrosulfonamide and GSK872 (n ≥, 6 statistical analyses by two-tailed paired t-test). **(F–H)** Analyses of impact of inhibitors of TLR4 engagement and signalling; inflammasome formation and the RIPK1-3 pathway broken down according to the HAdV species/type. (n ≥ 3, statistical analyses by two-tailed paired t-test).
