# Supplementary material for: Lactoferrin Retargets Human Adenoviruses to TLR4 to Induce an Abortive NLRP3-Associated Pyroptotic Response in Human Phagocytes
Source: Front Immunol. 2021 May 20;12:685218. doi: 10.3389/fimmu.2021.685218 (PMC8173049; doi:10.3389/fimmu.2021.685218)
Supplement: Supplementary Figure 1 — Analyses of lactoferrin binding to HAdV-C5, -D26 and -B35 by SPR (A) HAdV-C5, -D26, and -B35 were covalently coupled to a CM5 sensor chip and escalating doses of lactoferrin (6.25 - 200 nM) for KD determination. Depicted are overlaid sensorgrams (RU = resonance units); (B) The association and dissociation rates of lactoferrin for HAdV-C5, -D26 and -B35 capsids (C) Representative flow cytometry profiles of cells incubated with HAdVs ± lactoferrin. DCs were mock-treated (grey), incubated with HAdV -C5, -D26 and -B35 alone (red), with lactoferrin complexed with HAdV (blue), with HAdV for 30 min and then lactoferrin (green) or with lactoferrin for 30 min and then HAdV (purple). Fluorescence was analysed 24 h post-incubation; (D) monocytes and (E) LCs were incubated with HAdVs ± lactoferrin and fluorescence was analysed 24 h post-incubation (n = 5, statistical analyses by two-tailed paired t-test). [file Image_1.pdf]

FIGURE S1

A

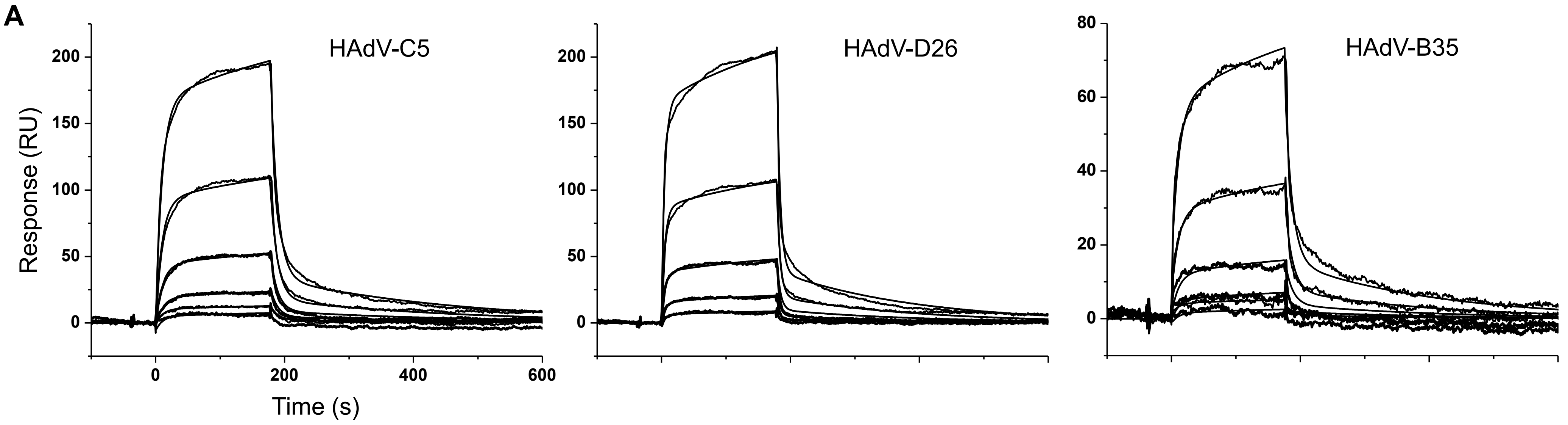

B

Virus	ka1 (1/Ms)	kd1 (1/s)	ka2 (1/s)	kd2 (1/s)	KD (M)
HAdV-C5	8.1×10^{4}	9.1×10^{-2}	1.4×10^{-3}	3.5×10^{-3}	8.0×10^{-7}
HAdV-D26	5.3×10^{4}	1.9×10^{-1}	1.9×10^{-3}	4.7×10^{-3}	2.6×10^{-6}
HAdV-B35	1.0×10^{3}	8.9×10^{-2}	2.5×10^{-3}	4.5×10^{-3}	5.4×10^{-5}

C

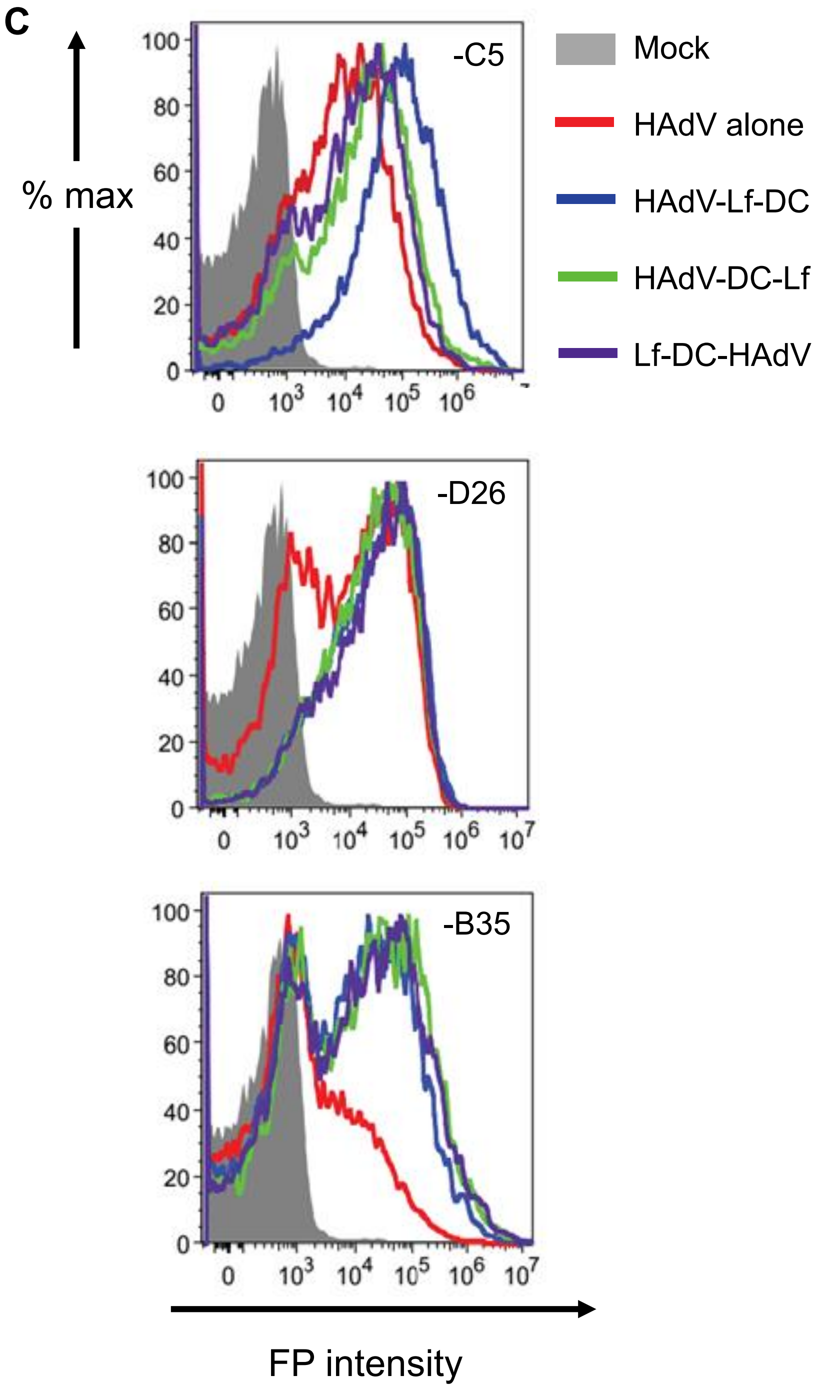

D

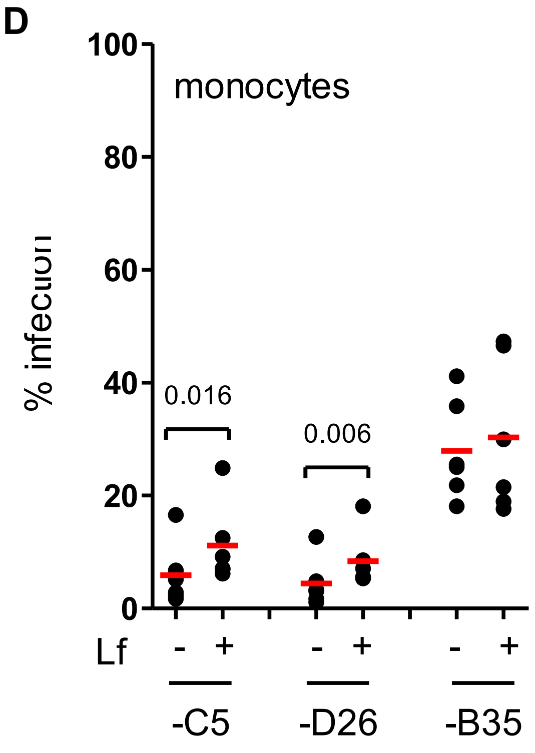

E

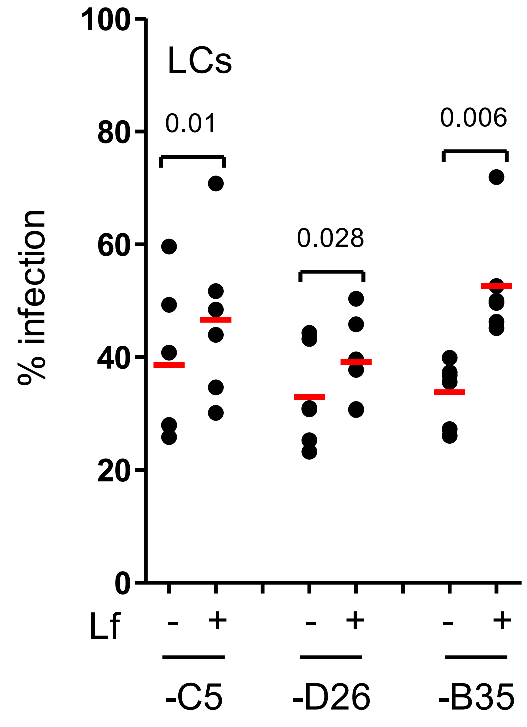

FIGURE S2

A

cytokines	mock	-C5	-D26	-B35	Lf	-C5 + Lf	-D26 + Lf	-B35 + Lf	LPS
CCL2	25.6	31.3	568	744	892	1198	1053	1432	250
CXCL1	168	180	510	527	5605	7381	7016	7878	8407
CXCL2	75.2	82.2	164	179	1110	2415	3632	4607	3457
CXCL5	193	196	309	300	1165	1363	1353	1397	1528
CXCL8	54.5	67.4	461	321	3414	5187	4589	6010	6998
CCL15	122	121	373	560	643	742	777	853	872
CCL20	4.57	6.57	31.7	24.3	599	795	779	851	866
CCl24	5376	5428	5142	4935	4618	5524	5163	5481	5603
TRAIL	23.5	6.59	48.6	249	79.8	79.8	178	249	89.2
CXCL12	141	152	347	392	586	696	677	845	775
IL-7	90.6	82.1	90.6	181	98.5	106	212	246	226
CXCL9	95.4	140	2476	4976	2773	3940	5390	6281	6853
CCL3	35.3	47.1	485	830	1061	1506	1378	1460	1782
IL12-p40	112	107	112	214	174	295	331	985	948
CXCL11	4.46	8.71	236	431	521	640	679	861	789
CXCL10	164	534	7817	8106	6735	9129	8318	9112	10132
CCL5	88.8	75.7	116	548	905	1231	1868	4470	6528
IL-1α	3.97	3.83	5.78	9.25	10.8	52.3	257	797	170
IL-1β	0.43	0.69	16.6	4.58	23.5	291	788	2317	116
IL-6	24.7	58.5	1160	1427	3554	5268	4991	5468	6428
TNF	65.2	109	998	1437	2417	4256	4438	4714	4879
IFN-β	65.5	85.5	1471	2456	191	1181	3884	3459	4234

B

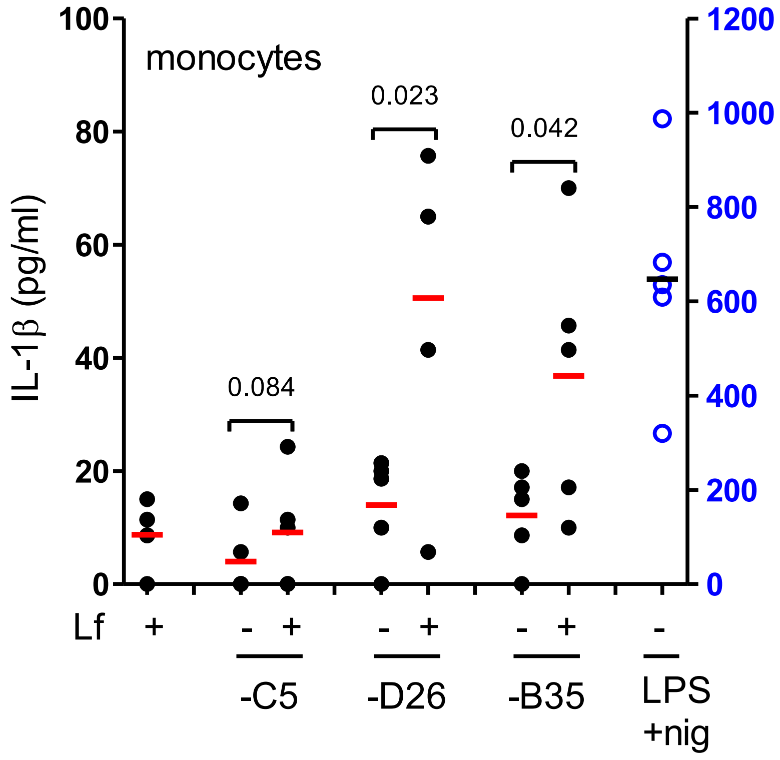

C

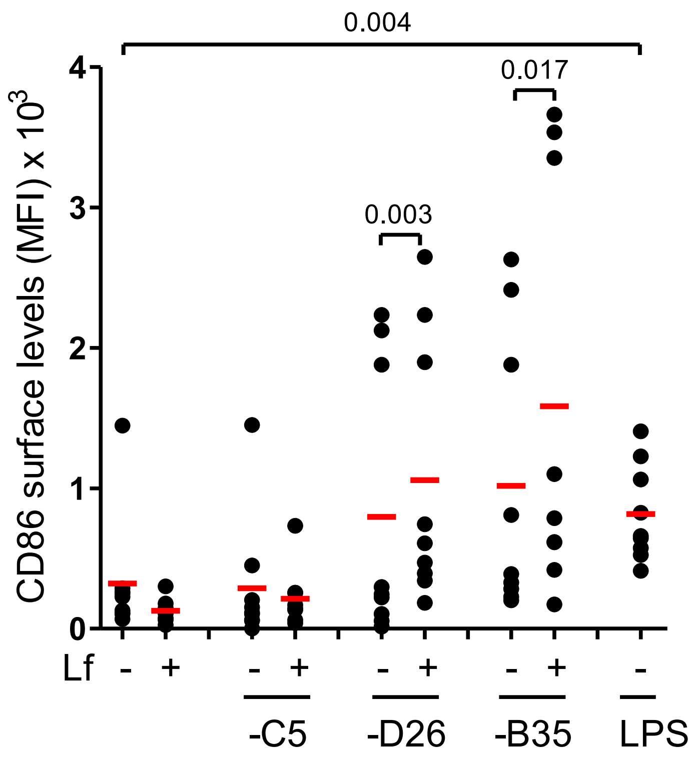

D

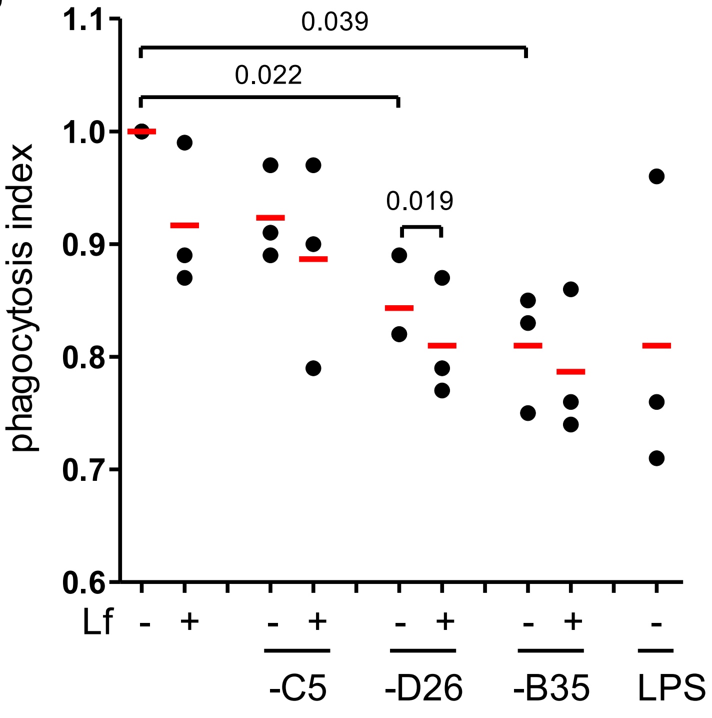

FIGURE S3

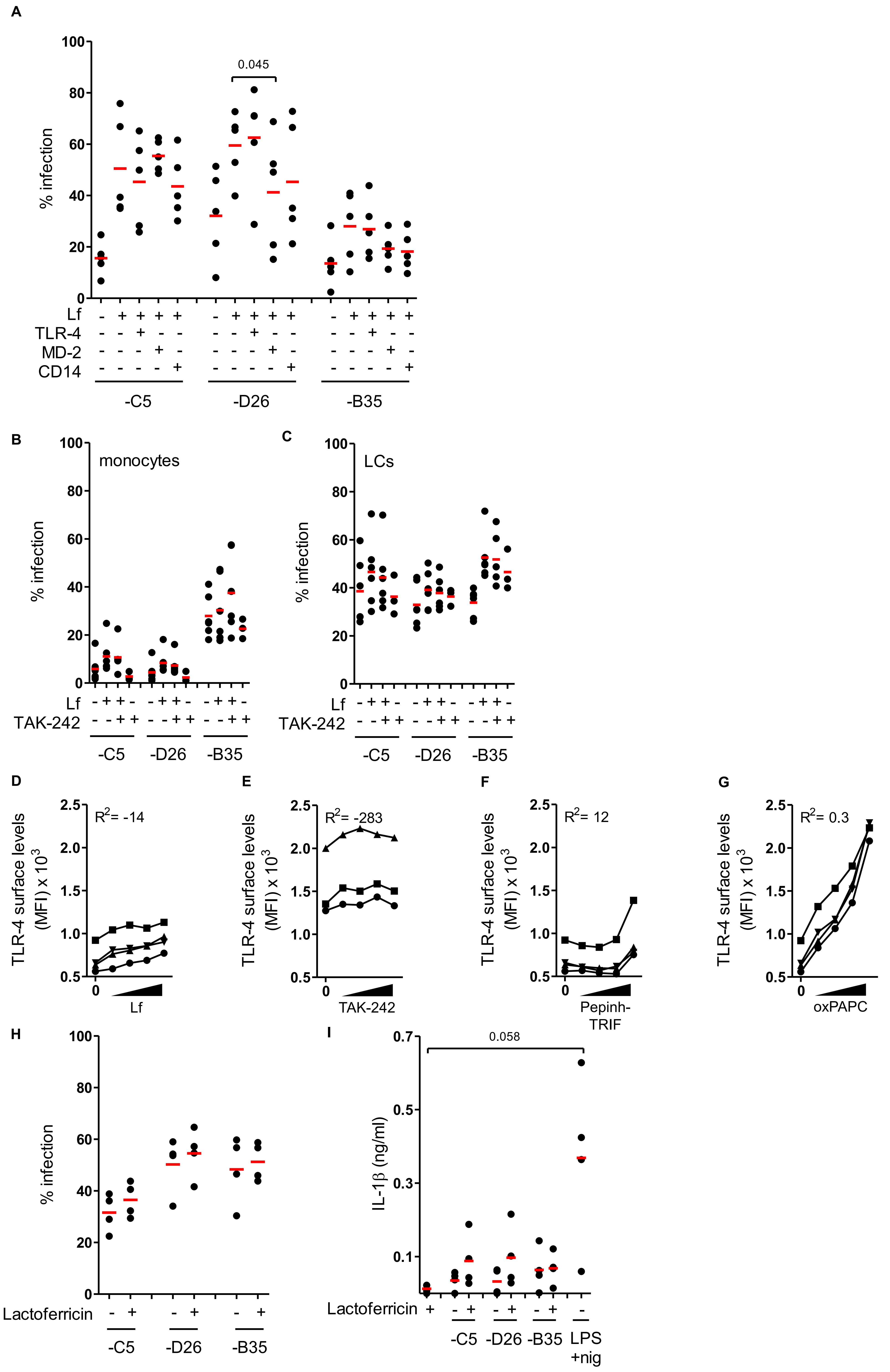

A
% infection
0.045
Lf
TLR-4
MD-2
CD14
-C5
-D26
-B35
B
monocytes
% infection
Lf
TAK-242
-C5
-D26
-B35
C
LCs
% infection
Lf
TAK-242
-C5
-D26
-B35
D
R2= -14
TLR-4 surface levels (MFI) x 10^3
Lf
E
R2= -283
TLR-4 surface levels (MFI) x 10^3
TAK-242
F
R2= 12
TLR-4 surface levels (MFI) x 10^3
Pepinh-TRIF
G
R2= 0.3
TLR-4 surface levels (MFI) x 10^3
oxPAPC
H
% infection
Lactoferricin
-C5
-D26
-B35
I
0.058
IL-1β (ng/ml)
Lactoferricin
-C5
-D26
-B35
LPS +nig

FIGURE S4

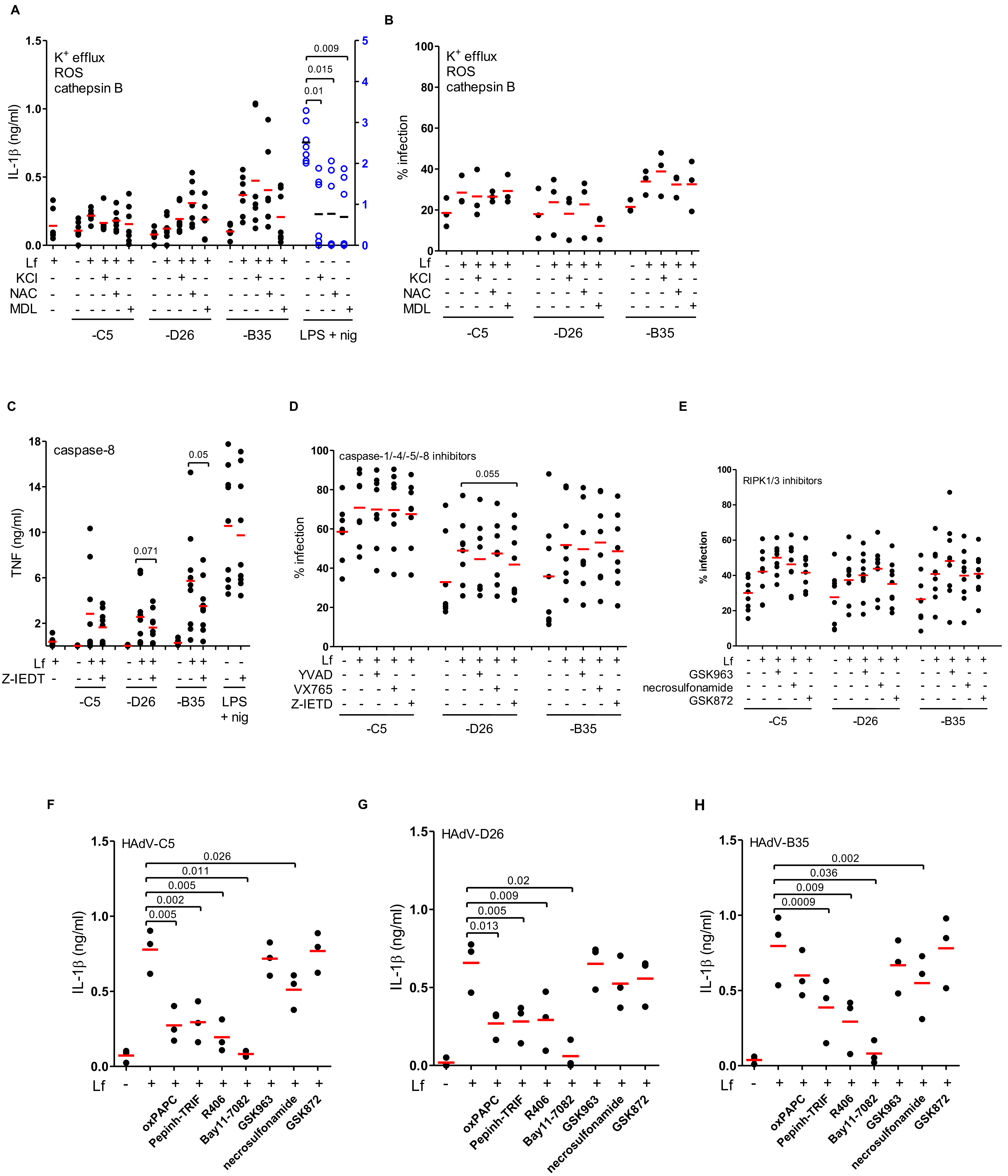

A
K+ efflux
ROS
cathepsin B
IL-1β (ng/ml)
0.009
0.015
0.01
Lf
KCl
NAC
MDL
-C5
-D26
-B35
LPS + nig
B
% infection
-C5
-D26
-B35
C
caspase-8
TNF (ng/ml)
0.071
0.05
Z-IEDT
LPS + nig
D
caspase-1/-4/-5/-8 inhibitors
% infection
0.055
YVAD
VX765
Z-IETD
E
RIPK1/3 inhibitors
% infection
GSK963
necrosulfonamide
GSK872
F
HAdV-C5
0.026
0.011
0.005
0.002
0.005
oxPAPC
Pepinh-TRIF
R406
Bay11-7082
GSK963
necrosulfonamide
GSK872
G
HAdV-D26
0.02
0.009
0.005
0.013
H
HAdV-B35
0.002
0.036
0.009
0.0009
